# Does the second messenger cAMP have a more complex role in controlling filamentous fungal morphology and metabolite production?

**DOI:** 10.1002/mbo3.627

**Published:** 2018-04-06

**Authors:** Ramkumar B. Nair, Rebecca Gmoser, Patrik R. Lennartsson, Mohammad J. Taherzadeh

**Affiliations:** ^1^ Swedish Centre for Resource Recovery University of Borås Borås Sweden; ^2^ Mycorena AB, Stena Center 1 A Gothenburg Sweden

## Abstract

The effect of second messenger cAMP on the physiological aspects of fungal cells such as pigmentation has been reported previously. However, their actual role in the cellular biochemical cascade that eventually affects the fungal growth morphology, such as mycelial pellet formation, is unclear. This article intends to open up the detailed study on the possible correlative effect of cAMP on the morphological and physiological growth aspects of filamentous fungi, with special emphasis on the industrial metabolite production.

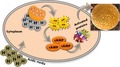

## 1 SUMMARY

Over many decades, filamentous fungi have been used for industrial production of commercially relevant products such as enzymes, antibiotics, feed products, organic acids, and many others. The wide application of filamentous fungi is generally related to their metabolic diversity giving rise to a panoply of products, their ability to grow on substrates of different complexity, and their tradition to produce products for human consumption giving many of them the status of GRAS (generally recognized as safe) (Finkelstein, 1992). Thus, filamentous fungi have a high potential for being included in new or established biorefineries and can be used for the valorization of waste streams from existing industrial facilities. For the last two decades, extensive research has been made by our research group to explore the potential of using edible filamentous fungi from both zygomycetes and ascomycetes fungal strains for valorization of industrial and agricultural waste streams (Bátori, Ferreira, Taherzadeh, & Lennartsson, 2015; Ferreira, Lennartsson, & Taherzadeh, 2014; Mahboubi, Ferreira, Taherzadeh, & Lennartsson, 2017; Nair, Lundin, Brandberg, Lennartsson, & Taherzadeh, 2015; Sues, Millati, Edebo, & Taherzadeh, 2005; Taherzadeh, Fox, Hjorth, & Edebo, 2003). Particularly, the ascomycete, *Neurospora intermedia* (the fungus used for the preparation of the Indonesian food *Oncom)*, has recently been developed as a potential substitute for high protein animal feed or aquatic feed and also as an ethanol producer from various industrial waste streams (Nair & Taherzadeh, 2016). Exploring its ability to grow rapidly on various types of industrial waste streams, *N. intermedia* could become a core biocatalyst in any biorefinery approach with its potential to produce an array of products (Gmoser, Ferreira, Lennartsson, & Taherzadeh, 2017). For the first time, our group has recently manipulated *N. intermedia* to grow as mycelial pellets (Nair, Lennartsson, & Taherzadeh, 2016), thereby enabling it to adopt diverse morphologies when cultivated in submerged cultures, such as the uniform and long filaments or entangled filaments in pellets or clumps. Filamentous fungal morphology has been reported to greatly influence the secretion of several metabolites and products (Choudhari, Ananthanarayan, & Singhal, 2008; Torres et al., 2016). A high fungal concentration with entangled mycelia or filaments results in a highly viscous suspension with non‐Newtonian properties, which reduces the homogeneity of nutrition, temperature, oxygen, and other parameters. Growth in the form of dense pellets generates a less viscous medium with Newtonian properties, but the internal mass transfer rate is limited by pellet size and compactness. These changes in gas–liquid mass transfer can thus affect the formation and secretion of products or metabolites (Torres et al., 2016).

Experiments in our lab with *N. intermedia* pellets (results yet to be published) indicate that there is a link between pellet formation and pigmentation. Culture factors resulting in evenly distributed small pellets in the liquid medium gave rise to a higher pigment accumulation in the biomass compared to mycelial and big clumps. Since the production of secondary metabolites, for example, pigments has been shown to be a stress‐related response (Yu & Keller, 2005), it could possibly have an interaction with other stress‐induced morphological response of the cells, such as mycelial pellet formation. Considering the pigment formation in filamentous fungi in general, it could reveal a significant correlation between the metabolite production to the molecular‐level cell response such as the production of the second messenger cyclic adenosine monophosphate (cAMP). Cyclic adenosine monophosphate works as an important component of the signal transduction pathway regulating cellular responses and has been shown to control a variety of functions in fungal cells (Cohen, 1974; Pall, 1981). For example, previous studies have connected cAMP to morphological and developmental alterations in biotechnologically relevant fungal species, in which cAMP acts to activate or deactivate enzymes involved in branch initiation, tip wall growth or conidia (germination of asexual spores) formation by phosphorylating target proteins or protein kinases. The changes correlate with deletion or mutational activation of components of cAMP (*gna‐1,* coding for a Gα component of a heterotrimeric G complex). Also, mutation of a putative histidine kinase gene (*dcc‐1)* results in enhanced conidiation. Interestingly, this effect was reversed by exogenous cAMP addition, indicating DCC‐1 to be another part of the signaling pathway that promotes cAMP production (Avalos & Corrochano, 2013; Yang & Borkovich, 1999). Studies on *Fusarium graminearum* as well as on *Neurospora crassa* revealed that increased levels of exogenous cAMP causes a decrease in hyphal extention rate and an increase branching (Robson, Wiebe, & Trinci, 1991). Cyclic adenosine monophosphate has also been suggested to control conidial germination of the filamentous fungus *Aspergillus nidulans* in response to carbon source sensing (Fillinger, Chaveroche, Shimizu, Keller, & d'Enfert, 2002).

Only a few high‐quality research studies have been carried out so far to determine the effect of cAMP on microbial pigments as the key metabolite produced. Early studies on *N. crassa* have led a number of researchers to suggest that the level of exogenous cAMP showed a strong effect on the conidiation and pigmentation in *N. crassa* (Kritsky, Sokolovsky, Belozerskaya, & Chernysheva, 1982; Pall, 1981; Yang & Borkovich, 1999). Increased levels of cAMP suppress the secondary metabolite pathway, observed, for example, in *Monascus* (lovastatin and red pigments). In this study, lovastatin and pigment production was downregulated when an excess amount of glucose was available in the medium, in connection with cAMP production (Miyake, Zhang, Kono, Nozaki, & Sammot, 2006). The hypothesis was also supported by Murayama, Uno, Hamamoto, and Ishikawa (1985) and García‐Martínez, Ádám, and Avalos (2012) using mutant *N. crassa* and *Fusarium fujikuroi,* respectively. Mutant cells with defective *acyA* gene (the gene coding for adenylate cyclase that generate cAMP), resulting in lower levels of intracellular cAMP have been observed to enhance pigment production compared to the wild‐type cells (García‐Martínez et al., 2012; Murayama et al., 1985). The relationship between cAMP level and the accumulation of pigments can be strongly dependent on the route of the cAMP signal that could further activate the enzymes downstream. However, little is known about the influence of cAMP level and its crucial role in determining the morphological states such as the pellet formation in filamentous fungi in general.

**Figure 1:**
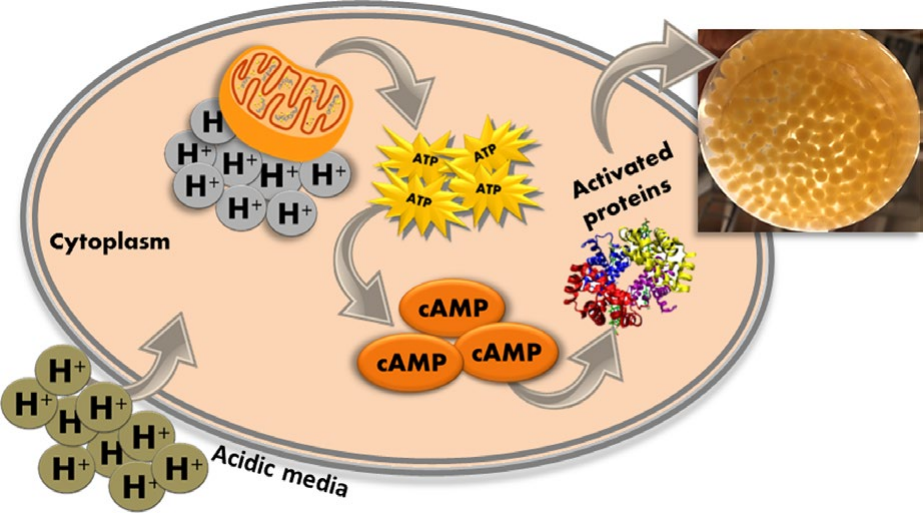
Cellular mechanism of pellet formation and pigmentation as a response to cyclic adenosine monophosphate (cAMP) synthesis in *Neurospora intermedia*. A high acidic conditions for the media (low pH) results in a electrochemical gradient leading to high ATP and high cAMP levels in the cell. The high levels of cAMP results in an activated cAMP receptor protein which leads to a series of cellular response, of which pigmentation and pellet formation are key responses in the case of *N. intermedia*. The detailed experimental results of this hypothesis are yet to be published

In the case of *N. intermedia*, subjected in our studies, the interesting aspect observed was that the level of cAMP in the cells influencing the pellet morphology also coincides with a high/low production of other valuable metabolites such as ethanol (results yet to be published). Hence, it could be hypothesized (Figure 1) that the factors that promote the pellet formation, mainly the media pH and carbon source (glucose as the critical factor), could be influencing the levels of cAMP as seen in our observations (data not shown) with glucose being previously reported to be a potent activator of cAMP synthesis (Rolland, Winderickx, & Thevelein, 2002). The cAMP pathway controls a wide variety of cellular properties in correlation with cellular proliferation. Miyake et al. (2006) suggested that carbon sources such as glucose repression might be largely dependent on the cAMP signaling pathway, which in turn represses the production of secondary metabolites in *Monascus*. Mapari, Meyer, and Thrane (2008) valuated morphology and yellow pigment production in *Epicoccum nigrum* and demonstrated that the growth‐type in form of pellet favored the production of yellow pigments, an observation that coincides with our research findings regarding ethanol production and pigmentation in *N. intermedia* (Méndez, Pérez, Montañéz, Martínez, & Aguilar, 2011; Nair et al., 2016). However, the influence of the levels of cAMP on fungal morphology such as mycelial pellets is yet to be investigated in detail and little is known about the amalgamated link between fungal morphological responses and the metabolite production, in relation to the factors affecting the cAMP levels in filamentous fungi. By regulating the factors affecting the cAMP level in the cell (such as media pH or carbon sources), the morphological form of the fungus and subsequently the metabolite production could be developed more favorably. This could potentially lead to better understanding of the fungal cellular responses that can eventually lead to the improvement or development of any metabolite (or product) at the existing or new filamentous fungi‐based industry or biorefinery.

To sum up our observations and hypothesis, we strongly believe that the insights on the correlation of the fungal morphology and metabolite production in relation to the factors affecting cAMP levels can consequently provide future avenues for improved metabolite (or product) formation from filamentous fungi with an industrial favorable growth morphology (pellet formation). Molecular‐level studies in line with the factors or responses controlling the cAMP levels in the fungal cell could be interesting aspects in the industrial scale studies for commercial metabolite production from filamentous fungi.

## ADDITIONAL REFERENCES FOR INDUSTRIAL APPLICATIONS OF *N. INTERMEDIA*

https://urn:nbn:se:hb:diva-674 (PhD thesis).

https://urn:nbn:se:hb:diva-12436 (PhD thesis).

## CONFLICT OF INTEREST

The authors declare that they have no conflict of interests.
